# Preparation of *Eleutherine americana*-Alginate Complex Microcapsules and Application in *Bifidobacterium longum*

**DOI:** 10.3390/nu7020831

**Published:** 2015-01-26

**Authors:** Atchara N Phoem, Suphitchaya Chanthachum, Supayang P Voravuthikunchai

**Affiliations:** 1 Department of Biology and Applied Biology, Faculty of Science and Technology, Songkhla Rajabhat University, Songkhla 90000, Thailand; E-Mail: aphoem@yahoo.com; 2 Department of Food Technology, Faculty of Agro-industry, Prince of Songkla University, Songkhla 90112, Thailand; E-Mail: Suphitchaya.c@psu.ac.th; 3 Department of Microbiology and Natural Product Research Center of Excellence, Faculty of Science, Prince of Songkla University, Songkhla 90112, Thailand

**Keywords:** sodium alginate, *Bifidobacterium longum*, *Eleutherine americana*, microencapsulation

## Abstract

Microencapsulation using extrusion and emulsion techniques was prepared for *Bifidobacterium longum* protection against sequential exposure to simulated gastric and intestinal juices, refrigeration storage and heat treatment. *Eleutherine americana* was used as the co-encapsulating agent. Hydrolysis of *E. americana* by gastric and intestinal juices was also determined. *E. americana* and its oligosaccharide extract demonstrated their resistance to low pH and partial tolerance to human α-amylase. Microencapsulated *B. longum* with *E. americana* and oligosaccharide extract prepared by the extrusion technique survived better than that by the emulsion technique under adverse conditions. Survival of microencapsulated cells after exposure to the juices and refrigeration storage was higher than free cells at Weeks 2 and 4. In addition, the viability of microencapsulated cells was better than free cells at 65 °C for 15 min. This work suggested that microencapsulated *B. longum* with *E. americana* offers the effective delivery of probiotics to colon and maintains their survival in food products.

## 1. Introduction

Interest in functional foods containing probiotics and/or prebiotics is increasing due to their potential role in health promoting and disease preventing properties. Probiotics are live microorganisms that, when ingested in sufficient numbers, can confer beneficial effects to host. Maintenance of the survival of probiotics until they reach the gut is one of the factor requirements for the health promoting properties. It has been recommended that the viability of probiotics should be at least 10^7^ cfu/g of the food product at the time of consumption [1]. Several factors have been claimed to affect the viability of probiotics, including heat processing [2], storage temperature [3] and gastrointestinal conditions [4]. Bifidobacteria belonging to the dominant infant gut microbiota are known as commonly-selected probiotics. These problems have encouraged researchers to search for new techniques to improve their viability.

In recent years, microencapsulation via extrusion and emulsion techniques has been applied for the protection of probiotics against gastrointestinal conditions and food products. The most commonly employed matrix for microencapsulation is sodium alginate, a natural heteropolysaccharides of d-mannuronic and l-guluronic linked with a glycosidic bond. However, sodium alginate disintegrates in harsh chemical environments [5]. To overcome these problems, a combination of sodium alginate with prebiotics produces beads with good stability, resulting in the development of probiotic viability in harsh conditions [6]. It has previously been reported that microencapsulation of probiotics with pectin [7] and chitosan [8,9] was able to increase their survival under gastrointestinal conditions and refrigeration storage. Prebiotic oligosaccharides are found in many sources, including fruits, vegetables, milk and honey. A major challenge in relation to the application of oligosaccharide as a co-encapsulating agent is its resistance to the gastrointestinal transit. Only a few studies have been reported on the hydrolysis of oligosaccharides by simulated gastrointestinal conditions [10,11]. However, mixed oligosaccharides were resistant to hydrolysis by simulated gastric juice and α-amylase [12].

*Eleutherine americana* Merr. is an herbal plant whose red bulb has been used as folk medicine. There have been a number of studies on *E. americana* bulb extract for applications in food [13,14]. Furthermore, both the extract and oligosaccharide extract have been used as prebiotics to produce growth stimulation on beneficial bacteria, which resulted in an increase in the production of short chain fatty acids [15]. However, there are no reports available on the application of *E. americana* as a co-encapsulating agent in protecting probiotic bacteria against gastrointestinal conditions.

Therefore, the objectives of this study were to evaluate the enhanced survival of microencapsulated *B. longum* with *E. americana* extract and oligosaccharide extract prepared by extrusion and emulsion techniques in gastrointestinal conditions and food products.

## 2. Materials and Methods

### 2.1. Probiotic Bacteria from Infant Feces

A potential probiotic bacteria, *Bifidobacterium longum*, isolated from healthy infant feces [16] in our previous study was used as the target strain in this present work.

*B. longum* cells were cultivated in 50 mL of MRS broth (de Man, Rogosa and Sharpe, Merck, Damstadt, Germany) supplemented with 0.05% (w/v) L-cysteine hydrochloride and incubated at 37 °C under anaerobic conditions for 24 h. The cultures were grown and transferred three times before use. The cell pellets were obtained from 24-h old culture by centrifugation at 10,000× *g* for 20 min, at 4 °C and washed twice with 0.85% (w/v) pre-reduced normal saline solution. Cell pellets were re-suspended in 10 mL of 0.1% (w/v) pre-reduced peptone solution and adjusted to reach a final cell density of approximately 1 × 10^10^ cfu/mL. They were divided into 2 parts: one part was used for microencapsulation and the other as free cells.

### 2.2. Eleutherine americana Extract and Oligosaccharide Extract

Bulbs of *E. americana* were collected from Songkhla, Thailand. Classified reference voucher specimens were deposited at the Herbarium of the Faculty of Pharmaceutical Sciences, Prince of Songkla University, Hat Yai, Songkhla, Thailand. The extract was obtained through a hot water extraction method [15] and filtered with 125-mm Whatman filter paper (Whatman Int. Ltd., Maidstone, UK) under vacuum at room temperature. The filtrates were dried by a freeze dryer (Flexi Dry, Munich, Germany).

*E. americana* extract was partially purified using *Saccharomyces cerevisiae* BCC 12652 to remove reducing sugar [15]. The extract was sterilized by filtration through a 0.22-μm membrane, followed by precipitation twice by 80% ethanol at 4 °C for 12 h. The sample was centrifuged at 10,000× *g* for 20 min, at 4 °C to remove the supernatant, and the attained supernatant was completely dried. Commercial fructo-oligosaccharide (Sigma-Aldrich, Steinheim, Germany) linked with a β-2,1 bond between the fructose monomer units was used as the reference. The extract, oligosaccharide extract and commercial fructo-oligosaccharides were dissolved in sterile distilled water and used for further studies.

### 2.3. Assessment of Eleutherine americana Stability after Sequential Incubation in Simulated Human Gastric and Intestinal Juices

*E. americana* extract and oligosaccharide extract were tested for gastric juice resistance, compared with commercial fructo-oligosaccharides as a prebiotic reference, according to the method of Korakli and co-workers [17]. A one percent solution was prepared by dissolving the substrates in sterile distilled water. Simulated human gastric juice was mimicked by hydrochloric acid (HCl) buffer containing: NaCl, 8 g; KCl, 0.2 g; Na_2_HPO_4_.2H_2_O, 8.25 g; NaH_2_PO_4_, 14.35 g; CaCl_2_.2H_2_O, 0.1 g; MgCl_2_.H_2_O, 0.18 g; and pepsin (Sigma-Aldrich), 3 g/L; and the pH was adjusted to 2, 3, 4 and 5 [12]. One milliliter of the sample was added to 9 mL of HCl buffer of all of the pH conditions, and the reaction mixture was incubated at 37 °C for 0, 2, 4 and 6 h. Reducing sugar and total sugar in the samples were determined by the dinitrosalicylic acid method [18] and the phenol-sulfuric acid method [19], respectively. The percentage of hydrolysis of the samples was calculated by the following equation (1).

(1) $$\text{Hydrolysis}\;\left(\%\right)=\frac{\text{reducing}\;\text{sugar}\;\text{released}}{\text{total}\;\text{sugar}\;\text{content}-\text{initial}\;\text{reducing}\;\text{sugar}\;\text{content}}\times 100\%$$

The reaction in HCl buffer at pH 2 for 6 h was stopped by adding 1 M NaOH to pH 7. The simulated intestinal juice, pH 5, 6, 7 and 8, containing the following—NaCl, 6.50 g; KCl, 0.84 g; CaCl_2_, 0.22 g; NaHCO_3_, 1.39 g; bile salt (Difco, Dickinson, MI, USA), 3 g; and α-amylase (Sigma-Aldrich), 1 g/L—was added to the sample and incubated at 37 °C for 0, 2, 4 and 6 h. The percentage of hydrolysis of the sample was calculated by the equation as described above.

### 2.4. Microencapsulation of Bifidobacterium longum with Eleutherine americana

#### 2.4.1. Microencapsulation Procedures

The extrusion technique was performed using a modified microencapsulation process of Krasaekoopt and co-workers [20]. Two milliliters of the cell suspension (1 × 10^9^ cfu/mL) were mixed with 16 mL of sterile 2% (w/v) sodium alginate solution (Fluka, Switzerland) and separately added with 1% (w/v) of *E. americana* extract, oligosaccharide extract, commercial fructo-oligosaccharides and distilled water (without the co-encapsulating agents). Then, the mixture was injected through a syringe needle size 23G (Nipro, Japan) into sterilized 0.1 M CaCl_2_ solution (Difco) and allowed to harden for 30 min in CaCl_2_ solution. Beads were washed twice with 0.85% (w/v) pre-reduced normal saline solution and stored in 0.1% (w/v) pre-reduced peptone solution (pH 6) at 4 °C until use. The free cells were used as the control. The bead sizes were determined by measuring the diameters of 100 beads using a vernier caliper.

The emulsion technique was performed using a modified method of Brinques and Ayub [8]. Four milliliters of the cell suspension (1 × 10^9^ cfu/mL) were mixed with 32 mL of sterile 2% (w/v) sodium alginate solution and supplemented with 4 mL of 1% (w/v), the final concentration of *E. americana* extract, oligosaccharide extract, commercial fructo-oligosaccharides and distilled water (without the co-encapsulating agents). The mixture was added dropwise to 100 mL of vegetable oil containing Tween 80 (0.1% v/v, Merck) and stirred at 200 rpm of magnetic stirring for 10 min. One hundred and fifty milliliters of 0.1 M CaCl_2_ solution were added gently down the side of the beaker until the water/oil emulsion was broken. After 30 min, the beads were removed from the aqueous phase, washed with 0.85% (w/v) pre-reduced normal saline solution and stored at 4 °C until use. The free cells were used as the control. The particle size of the beads was measured using a particle size analyzer (LPSA, LS230, Coulter, CA, USA) according to Su and co-workers [21]. One gram of beads was transferred to a sample holder containing 500 mL of distilled water, and the measurement was performed by static light scattering.

#### 2.4.2. Efficacy of Entrapment and Release of Microencapsulated *Bifidobacterium longum*

One gram of beads was added to 9 mL of 0.1 M pre-reduced phosphate buffer, pH 7.4, followed by homogenization in a stomacher for 5 min. The samples were then centrifuged at 10,000 × *g* for 10 min, at 4 °C. The supernatant was serially diluted with normal saline solution and plated on MRS agar modified with the addition of 0.05% (w/v) l-cysteine hydrochloride. The plates were incubated at 37 °C under anaerobic conditions for 48 h. After incubation, viable cells were counted and expressed as log colony-forming units per gram (log_10_ cfu/g). The encapsulation yield, which is a combined measurement of the efficacy of entrapment and the survival of viable cells during the microencapsulation procedure, was calculated as proposed by Chavarri and co-workers [9].

Encapsulation yield (%) = N/No × 100 (2)

where N is the number of viable entrapped cells released from the beads and No is the number of free cells added to the biopolymer mix during the formation of the beads.

For quantitative measurements of cell viability, the beads were solubilized to release the microencapsulated cells [22]. One gram of beads was added to 9 mL of 0.1 M pre-reduced phosphate buffer (pH 7.4) followed by incubation at 37 °C under anaerobic conditions for 1 h. The number of viable cells in the suspension was then determined by a plate count, as described in Section 2.4.2.

Cell release (%) = N/No × 100 (3)

where N is the number of viable cells in the suspension released from the beads and No is the number of free cells added to the biopolymer mix during the formation of the beads.

### 2.5. Survival of Microencapsulated Bifidobacterium longum after Sequential Incubation in Simulated Human Gastric and Intestinal Juices

Microencapsulated *B. longum* and free cells were stored in 0.1% (w/v) pre-reduced peptone solution, (pH 6) at 4 °C for 0, 2 and 4 weeks, and the survival of microencapsulated *B. longum* after exposure to simulated gastric and intestinal juices was analyzed. The HCl buffer of pH 2 containing pepsin 3 g/L was used to simulate gastric juice, as described by Sandoval-Castilla and co-workers [7]. The microencapsulated *B. longum* (1 g) and free cells (1 mL) were added to 9 mL of simulated gastric juice and incubated at 37 °C under anaerobic conditions for 3 h. The samples were transferred to 0.1 M pre-reduced phosphate buffer (pH 7.4) followed by homogenization in a stomacher for 5 min. The viable cell count was calculated as described in Section 2.4.2. The samples that were exposed to simulated gastric juice were centrifuged, washed with 0.85% (w/v) pre-reduced normal saline solution and added to 9 mL of simulated intestinal juice (pH 7.4) containing: NaCl, 6.50 g; KCl, 0.84 g; CaCl_2_, 0.22 g; NaHCO_3_, 1.39 g; l-cysteine hydrochloride, 0.50 g; bile salt, 3 g; and α-amylase, 1 g/L. They were anaerobically incubated at 37 °C for 3 h. The samples were centrifuged, washed with normal saline solution and the viable cells counted, as described in Section 2.4.2.

### 2.6. Survival of Microencapsulated Bifidobacterium longum under Refrigeration Storage and Heat Treatment

#### 2.6.1. Resistance to Refrigeration Storage

The viability of microencapsulated *B. longum* cells in refrigeration storage was studied by modifying the method of Brinques and Ayub [8]. Briefly, microencapsulated *B. longum* (1 g) and free cells (1 mL) were added to 9 mL of 0.1% (w/v) pre-reduced peptone solution with pH 4 and 6. The samples were kept in the refrigerator at 4 °C for 0, 2 and 4 weeks. They were used for the viable cell count after depolymerization in 0.1 M pre-reduced phosphate buffer, as described in Section 2.4.2.

#### 2.6.2. Resistance to Heat Treatment

The efficacy of microencapsulation in protecting viable cells during thermal process was assessed [23]. Microencapsulated *B. longum* (1 g) and free cells (1 mL) were added to 9 mL of 0.1% (w/v) pre-reduced peptone solution with pH 4 and 6. The samples were heated at 65 °C for 0, 15 and 30 min and cooled to room temperature (~25 °C). After depolymerization, the viable cell counts of *B. longum* were determined as described in Section 2.4.2.

### 2.7. Statistical Analysis

The data were reported as the mean ± the standard deviation (SD). Differences among groups were examined for statistical significance by one-way analysis of variance (ANOVA). The criterion for significance was *p* < 0.05.

## 3. Results and Discussion

### 3.1. Stability of Eleutherine americana after Sequential Exposure to Simulated Human Gastric and Intestinal Juices

The hydrolysis pattern of *E. americana* extract, oligosaccharide extract and commercial fructo-oligosaccharides after incubation in simulated human gastric juice at 2, 4 and 6 h are given in Table 1. The percentage of acid hydrolysis at pH 2 and 3 was significantly higher than pH 4 and 5 (*p* < 0.05). The degree of hydrolysis increased with the decreasing pH of the juice. There was a high proton concentration at very low pH [10]. The oligosaccharide extract significantly demonstrated the highest acid resistance, compared with the extract and commercial fructo-oligosaccharides (*p* < 0.05). However, there was no significant difference in acid resistance between the extract and commercial fructo-oligosaccharides (*p* > 0.05). The maximum hydrolysis of the oligosaccharide extract at pH 2, 3, 4 and 5 was 7.51%, 7.42%, 3.47% and 3.12%, respectively, within 6 h. Food is usually retained in the human stomach for 2–6 h, where gastric juice at pH 2–5 is released [24]. Therefore, when the oligosaccharide extract is consumed, 93% of it is estimated to reach the small intestine. Similarly, other workers have documented that *Mangifera pajang* fibrous polysaccharides [10] and mixed oligosaccharides [12] were resistant to hydrolysis by simulated gastric juice. Starch [25] has also been reported to be highly resistant to acidic conditions in an animal model.

The degree of hydrolysis of the substrates after sequential exposure to simulated gastric and intestinal juices increased with incubation time at 2, 4 and 6 h (Table 1). The degree of hydrolysis at pH 6, 7 and 8 was significantly higher than pH 5 (*p* < 0.05). However, there were no significant differences at pH 6, 7 and 8 (*p* > 0.05). The oligosaccharide extract showed the highest α-amylase resistance, compared with the extract and commercial fructo-oligosaccharides, which gave a maximum hydrolysis of 12.15%, 22.53%, 22.21% and 22.37% at pH 5, 6, 7 and 8, respectively, within 6 h. *E. americana* extract and the oligosaccharide extract were resistant to hydrolysis, and they were still intact during exposure to simulated intestinal juice at pH 5–8 for 6 h. The degree of hydrolysis enhanced with the increasing pH of the juice. Simulated intestinal juice at pH 6, 7 and 8 was suitable for enzymatic activity. Normally, carbohydrates are hydrolyzed in the small intestine, which gave hydrolysis of 30%. Isomaltase, glucoamylase, maltase, sucrase and lactase digest the carbohydrate at α-1,4- and α-1,6-linked glucosaccharides in the small intestine and yield monosaccharides as end products [26]. The small enzymatic hydrolysis of oligosaccharide extract might be due to the presence of β and α-glycosidic linkages in the oligosaccharide extract. It was estimated that 78% of the oligosaccharide extract consumed would reach the colon. Similarly, *Mangifera pajang* fibrous polysaccharides [10] and mixed oligosaccharides [12] were resistant to hydrolysis by human α-amylase. 

**Table 1:** Hydrolysis (%) of *Eleutherine americana* after sequential exposure to simulated human gastric and intestinal juices.

Substrates Time (h)		Hydrolysis (%) ^1^				Hydrolysis (%) ^1^			
		Simulated Gastric Juice pH				Simulated Gastric and Intestinal Juices pH			
		2	3	4	5	5	6	7	8
*E. americana* extract	0	0.57 ± 0.11 ^aA^	0.57 ± 0.05 ^aA^	0.55 ± 0.01 ^aA^	0.55 ± 0.04 ^aA^	0.82 ± 0.11 ^aA^	0.80 ± 0.03 ^aA^	0.79 ± 0.01 ^aA^	0.82 ± 0.03 ^aA^
Oligosaccharide extract		0.37 ± 0.18 ^aA^	0.38 ± 0.15 ^aA^	0.35 ± 0.05 ^aA^	0.34 ± 0.14 ^aA^	0.71 ± 0.13 ^aA^	0.75 ± 0.08 ^aA^	0.73 ± 0.02 ^aA^	0.70 ± 0.21 ^aA^
Commercial fructo-oligosaccharides		0.56 ± 0.21 ^aA^	0.58 ± 0.20 ^aA^	0.54 ± 0.17 ^aA^	0.52 ± 0.16 ^aA^	0.78 ± 0.16 ^aA^	0.81 ± 0.12 ^aA^	0.75 ± 0.09 ^aA^	0.83 ± 0.14 ^aA^
*E. americana* extract	2	7.67 ± 0.10 ^aA^	7.73 ± 0.14 ^aA^	5.73 ± 0.21 ^aB^	5.71 ± 0.24 ^aB^	23.17 ± 0.20 ^aB^	31.14 ± 0.02 ^aA^	31.75 ± 0.10 ^aA^	31.51 ± 0.10 ^aA^
Oligosaccharide extract		5.80 ± 0.16 ^bA^	5.35 ± 0.08 ^bA^	1.78 ± 0.15 ^bB^	2.03 ± 0.06 ^bB^	12.07 ± 0.07 ^bB^	22.14 ± 0.16 ^bA^	22.17 ± 0.16 ^bA^	22.21 ± 0.08 ^bA^
Commercial fructo-oligosaccharides		7.95 ± 0.22 ^aA^	7.60 ± 0.03 ^aA^	5.75 ± 0.13 ^aB^	5.77 ± 0.17 ^aB^	23.21 ± 0.06 ^aB^	31.42 ± 0.15 ^aA^	31.73 ± 0.14 ^aA^	31.63 ± 0.11 ^aA^
*E. americana* extract	4	9.68 ± 0.10 ^aA^	9.60 ± 0.12 ^aA^	6.74 ± 0.08 ^aB^	6.81 ± 0.12 ^aB^	23.21 ± 0.25 ^aB^	31.30 ± 0.16 ^aA^	31.55 ± 0.15 ^aA^	31.45 ± 0.11 ^aA^
Oligosaccharide extract		7.62 ± 0.12 ^bA^	7.51 ± 0.08 ^bA^	3.54 ± 0.16 ^bB^	3.45 ± 0.05 ^bB^	12.17 ± 0.22 ^bB^	22.42 ± 0.24 ^bA^	22.32 ± 0.12 ^bA^	22.42 ± 0.16 ^bA^
Commercial fructo-oligosaccharides		9.75 ± 0.09 ^aA^	9.85 ± 0.14 ^aA^	6.69 ± 0.11 ^aB^	6.64 ± 0.10 ^aB^	23.09 ± 0.18 ^aB^	31.54 ± 0.17 ^aA^	31.61 ± 0.09 ^aA^	31.52 ± 0.09 ^aA^
*E. americana* extract	6	9.75 ± 0.07 ^aA^	9.68 ± 0.14 ^aA^	6.68 ± 0.04 ^aB^	6.74 ± 0.05 ^aB^	23.05 ± 0.03 ^aB^	31.37 ± 0.06 ^aA^	31.60 ± 0.21 ^aA^	31.58 ± 0.12 ^aA^
Oligosaccharide extract		7.51 ± 0.05 ^bA^	7.42 ± 0.15 ^bA^	3.47 ± 0.12 ^bB^	3.12 ± 0.09 ^bB^	12.15 ± 0.14 ^bB^	22.53 ± 0.04 ^bA^	22.21 ± 0.09 ^bA^	22.37 ± 0.15 ^bA^
Commercial fructo-oligosaccharides		9.86 ± 0.14 ^aA^	9.97 ± 0.04 ^aA^	6.67 ± 0.20 ^aB^	6.71 ± 0.14 ^aB^	23.15 ± 0.12 ^aB^	31.46 ± 0.11 ^aA^	31.68 ± 0.07 ^aA^	31.65 ± 0.04 ^aA^

Values are the means ± the standard deviation from duplicate determinations; different superscript uppercase letters (A, B) in the same row are significantly different (*p* < 0.05); different superscript lowercase letters (a, b) in the same column are significantly different among different substrates for each time (*p* < 0.05); ^1^ Hydrolysis (%) = reducing sugar released/total sugar content-initial reducing sugar content × 100%.

### 3.2. Properties of Microencapsulated Bifidobacterium longum with Eleutherine americana

The properties of microencapsulated *B. longum* with *E. americana* with respect to diameter, encapsulation yield (%) and cell release (%) are shown in Table 2. Microencapsulation via extrusion and emulsion techniques was applied for the combination of sodium alginate with the extract, oligosaccharide extract and commercial fructo-oligosaccharides, compared with alginate alone. High cell concentrations in the range of 9.37–9.67 log_10_ cfu/g beads were achieved in both co-encapsulated and encapsulated cells. There was no significant difference in diameter between co-encapsulated and encapsulated cells (*p* > 0.05). The type of co-encapsulating agents had no influence on the size of the beads. The diameter of beads made by the extrusion technique was 1.65–2.05 mm, which was significantly bigger than that of beads obtained through the emulsion technique (0.51–0.86 mm) (*p* < 0.05).

The encapsulation yield is a combined measurement of the efficiency of entrapment and the survival of *B. longum* during the microencapsulation techniques. The encapsulation yield of co-encapsulated cells was significantly higher than encapsulated cells (*p* < 0.05). However, the yield of co-encapsulated cells was similar in each co-encapsulating agent. There was a significant difference in the encapsulation yield between the extrusion and emulsion techniques (*p* < 0.05). The yield of co-encapsulated cells made by the extrusion and emulsion techniques was in the range of 93.34%–93.47% and 87.17%–87.48%, respectively. The combination of alginate with *E. americana* may reduce the porosity of the beads and decrease the leakage of the microencapsulated *B. longum*.

Co-encapsulating agents had no effect on cell release from the beads. The released cells from alginate beads made by the extrusion and emulsion techniques were 96.51% and 98.21%, respectively, within 1 h. *E. americana* was very suitable for application in a natural polymeric system, which could not delay the release of microencapsulated *B. longum.* The microencapsulated cells with *E. americana* were released by the mechanisms of degradation of the polymer network. The phosphate ions in the simulated colonic pH solution degrade the cross-linked matrix for the cell release [21]. Accordingly, microencapsulated *Bifidobacterium longum* BIOMA 5920 with human-like collagen [21], microencapsulated *Lactobacillus bulgaricus* with milk [27] and microencapsulated *Pediococcus acidilactici* with chitosan [28] were completely released in the simulated colonic pH solution within 1–2 h.

**Table 2:** Diameter, encapsulation yield (%) and cell release (%) of microencapsulated *Bifidobacterium longum* with *Eleutherine americana.*

Properties of Beads	Microencapsulation Techniques	Co-Encapsulated Cells			Encapsulated Cells
		Alginate-*E. americana* Extract	Alginate-Oligosaccharide Extract	Alginate-Commercial Fructo-Oligosaccharides	Alginate
Diameter (mm)	Extrusion technique	2.05 ± 0.18 ^aA^	1.87 ± 0.23 ^aA^	1.90 ± 0.31 ^aA^	1.65 ± 0.17 ^aA^
	Emulsion technique	0.86 ± 0.21 ^bA^	0.69 ± 0.12 ^bA^	0.67 ± 0.05 ^bA^	0.51 ± 0.18 ^bA^
Encapsulation yield (%) ^1^	Extrusion technique	93.34 ± 0.11 ^aA^	93.47 ± 0.24 ^aA^	93.38 ± 0.08 ^aA^	72.52 ± 0.24 ^aB^
	Emulsion technique	87.17 ± 0.09 ^bA^	87.32 ± 0.12 ^bA^	87.48 ± 0.17 ^bA^	67.34 ± 0.14 ^bB^
Cell release (%) ^2^	Extrusion technique	94.21 ± 0.16 ^bB^	94.15 ± 0.21 ^bB^	94.20 ± 0.04 ^bB^	96.51 ± 0.19 ^bA^
	Emulsion technique	96.41 ± 0.10 ^aB^	96.45 ± 0.08 ^aB^	96.32 ± 0.16 ^aB^	98.21 ± 0.22 ^aA^

Values are the means ± standard deviation from duplicate determinations; different superscript uppercase letters (A, B) in the same row are significantly different (*p* < 0.05); different superscript lowercase letters (a, b) in the same column are significantly different among different microencapsulation techniques for each bead property (*p* < 0.05); ^1^ Encapsulation yield (%) = N/No × 100, where N is the number of viable entrapped cells released from the beads and No is the number of free cells added to the biopolymer mix during the formation of the beads; ^2^ Cell release (%) = N/No × 100, where N is the number of viable cells in the suspension released from the beads and No is the number of free cells added to the biopolymer mix during the formation of the beads.

### 3.3. Viability of Microencapsulated Bifidobacterium longum with Eleutherine americana after Sequential Incubation in Simulated Gastric and Intestinal Juices

Survival of microencapsulated *B. longum* with *E. americana*, alginate alone and free cells after exposure to simulated gastric juice and simulated intestinal juice is shown in Table 3. The beads made by the extrusion and emulsion techniques were stored at 4 °C for two and four weeks. Initially, the number of viable *B. longum* was in the range of 9.37–9.67 log_10_ cfu/g beads. The number of viable cells reduced after refrigeration storage. The survival of encapsulated cells before and after sequential incubation in the acidic and enzymatic conditions was better than that of non-encapsulated cells at Weeks 0, 2 and 4 (*p* < 0.05). The formation of a hydrogel barrier by the compacted alginate layer was demonstrated to retard the permeation of simulated gastric and intestinal juices into the cells. Nevertheless, the alginate matrix at very low pH was documented to reduce in its molecular weight, causing a rapid degradation and release of the cells [27]. The viability of co-encapsulated cells before and after exposure to the simulated juices was higher than that of encapsulated cells at Weeks 2 and 4 (*p* < 0.05). Microencapsulated probiotics with carrageenan-locust bean gum-coated milk microspheres resulted in a reduction of the pore size, providing a more favorable anaerobic environment for the bacterial cells [27]. The number of co-encapsulated and encapsulated cells in the simulated gastric juice was higher than that of the simulated intestinal juice at four weeks, except that the live cell number of co-encapsulated *B. longum* with oligosaccharide extract was similar in both juices. The beads were much more stable in gastric conditions and broken when exposed to intestinal conditions, which facilitated the cell release from the gel layer into the intestinal system [20]. The highest survival of *B. longum* resulted from microencapsulation with the oligosaccharide extract. The reason might be the presence of β and α-glycosidic linkages in the oligosaccharide extract, which resist the activity of α-amylase in the simulated intestinal juice. The survival of *B. longum* microencapsulation with oligosaccharide extract made by the extrusion and emulsion techniques decreased from 9.15–8.17 and 8.17–7.27 log_10_ cfu/g, respectively, after sequential incubation in the simulated gastric and intestinal juices. The larger beads made by the extrusion technique may have afforded additional physical protection simply by enhancing the distance between encapsulated cells and the simulated juices [29]. However, none of the non-encapsulated cells survived after exposure to the simulated juices. In general, the acid tolerance of the cells depends on the pH profile of H^+^-ATPase and the composition of the cytoplasmic membrane of the cells [5].

**Table 3:** Survival of microencapsulated *Bifidobacterium longum* with *Eleutherine americana* after sequential incubation in simulated human gastric and intestinal juices.

Microencapsulation	Time (Week)	Plate Count (log_10_ cfu/g)					
		Pre-Gastric Juice		Post-Gastric Juice		Post-Intestinal Juice	
		Extrusion Technique	Emulsion Technique	Extrusion Technique	Emulsion Technique	Extrusion Technique	Emulsion Technique
Co-encapsulated cells: alginate-*E. americana* extract	0	9.57 ± 0.17 ^aA^	9.47 ± 0.22 ^aA^	9.42 ± 0.17 ^aA^	9.40 ± 0.23 ^aA^	9.72 ± 0.10 ^aA^	9.65 ± 0.16 ^aA^
Co-encapsulated cells: alginate-oligosaccharide extract		9.46 ± 0.11 ^aA^	9.37 ± 0.12 ^aA^	9.52 ± 0.27 ^aA^	9.59 ± 0.11 ^aA^	9.63 ± 0.15 ^aA^	9.60 ± 0.10 ^aA^
Co-encapsulated cells: alginate-commercial fructo-oligosaccharides		9.37 ± 0.05 ^aA^	9.45 ± 0.09 ^aA^	9.33 ± 0.16 ^aA^	9.55 ± 0.04 ^aA^	9.71 ± 0.11 ^aA^	9.62 ± 0.12 ^aA^
Encapsulated cells: alginate		9.57 ± 0.08 ^aA^	9.67 ± 0.01 ^aA^	9.53 ± 0.20 ^aA^	9.59 ± 0.17 ^aA^	9.53 ± 0.21 ^aA^	9.43 ± 0.06 ^aA^
Non-encapsulated cells: free cells		9.46 ± 0.13 ^aA^	9.46 ± 0.07 ^aA^	8.46 ± 0.18 ^bA^	8.46 ± 0.19 ^bA^	8.55 ± 0.16 ^bA^	8.55 ± 0.18 ^bA^
Co-encapsulated cells: alginate-*E. americana* extract	2	9.27 ± 0.19 ^aA^	8.34 ± 0.13 ^aB^	8.31 ± 0.06 ^aA^	7.31 ± 0.20 ^aB^	8.37 ± 0.05 ^aA^	7.23 ± 0.31 ^aB^
Co-encapsulated cells: alginate-oligosaccharide extract		9.17 ± 0.06 ^aA^	8.29 ± 0.09 ^aB^	8.34 ± 0.03 ^aA^	7.35 ± 0.06 ^aB^	8.13 ± 0.18 ^aA^	7.37 ± 0.23 ^aB^
Co-encapsulated cells: alginate-commercial fructo-oligosaccharides		9.08 ± 0.23 ^aA^	8.09 ± 0.11 ^aB^	8.21 ± 0.19 ^aA^	7.23 ± 0.16 ^aB^	8.03 ± 0.07 ^aA^	7.14 ± 0.05 ^aB^
Encapsulated cells: alginate		8.21 ± 0.12 ^bA^	7.26 ± 0.18 ^bB^	7.37 ± 0.14 ^bA^	6.42 ± 0.02 ^bB^	7.27 ± 0.11 ^bA^	6.23 ± 0.17 ^bB^
Non-encapsulated cells: free cells		4.68 ± 0.24 ^cA^	4.68 ± 0.21 ^cA^	2.35 ± 0.21 ^cA^	2.35 ± 0.13 ^cA^	2.47 ± 0.23 ^cA^	2.47 ± 0.12 ^cA^
Co-encapsulated cells: alginate-*E. americana* extract	4	8.22 ± 0.06 ^bA^	7.43 ± 0.16 ^bB^	7.15 ± 0.06 ^bA^	6.22 ± 0.21 ^bB^	6.31 ± 0.16 ^bA^	5.34 ± 0.22 ^bB^
Co-encapsulated cells: alginate-oligosaccharide extract		9.15 ± 0.15 ^aA^	8.17 ± 0.23 ^aB^	8.19 ± 0.17 ^aA^	7.18 ± 0.15 ^aB^	8.17 ± 0.12 ^aA^	7.27 ± 0.13 ^aB^
Co-encapsulated cells: alginate-commercial fructo-oligosaccharides		8.17 ± 0.14 ^bA^	7.16 ± 0.14^bB^	7.19 ± 0.11 ^bA^	6.26 ± 0.11 ^bB^	6.27 ± 0.17 ^bA^	5.24 ± 0.11 ^bB^
Encapsulated cells: alginate		6.31 ± 0.20 ^cA^	5.13 ± 0.10 ^cB^	5.62 ± 0.05 ^cA^	4.50 ± 0.07 ^cB^	4.45 ± 0.04 ^cA^	3.57 ± 0.06 ^cB^
Non-encapsulated cells: free cells		0.00 ± 0.00 ^dA^	0.00 ± 0.00 ^dA^	0.00 ± 0.00 ^dA^	0.00 ± 0.00 ^dA^	0.00 ± 0.00 ^dA^	0.00 ± 0.00 ^dA^

Values are the means ± the standard deviation from duplicate determinations; different superscript uppercase letters (A, B) in the same row are significantly different among different microencapsulation techniques for each gastrointestinal condition (*p* < 0.05); different superscript lowercase letters (a, b, c, d) in the same column are significantly different among different co-encapsulating agents for each time (*p* < 0.05).

### 3.4. Viability of Microencapsulated Bifidobacterium longum with Eleutherine americana under Refrigeration Storage and Heat Treatment

The effect of refrigeration storage on the survival of microencapsulated *B. longum* with *E. americana,* alginate alone and free cells is shown in Table 4. A high cell concentration in the range of 9.47–9.71 log_10_ cfu/g beads was produced by the extrusion and emulsion techniques and kept in peptone solution of pH 4 and 6 at 4 °C for two and four weeks. The live cell number of *B. longum* decreased after refrigeration storage. The number of viable cells in the peptone solution of pH 4 and 6 containing encapsulated cells was higher than that of non-encapsulated cells at Weeks 2 and 4 (*p* < 0.05). The formation of a hydrogel around the cell pellet was used for cell protection. This was because the peptone solution needed to permeate through the alginate layer before reaching the cells. The survival of co-encapsulated cells in the peptone solution was better than that of encapsulated cells (*p* < 0.05). There was a reduction of pore size, thus hindering the interaction between cells and the peptone solution [5]. *B. longum* in the peptone solution of pH 6 showed better survival than that of pH 4. The highest levels of viable *B. longum* cells were observed when microencapsulated with oligosaccharide extract at Week 4. The viability of *B. longum* microencapsulation with the oligosaccharide extract made by the extrusion and emulsion techniques was 9.21 and 8.23 log_10_ cfu/g, respectively, in the presence of the peptone solution of pH 6, and that of pH 4 was 8.60 and 7.52 log_10_ cfu/g, respectively. However, no survival of non-encapsulated cells was noted at four weeks. At low temperatures, enzyme activity in microorganisms becomes slower and reduces the fluidity of the cytoplasmic membrane, thus interfering with transport mechanisms [30]. Accordingly, microencapsulated probiotics with chitosan [6,9], human-like collagen [21] and starch [31] improved the viability compared to that of free cells under refrigeration at 4 °C for 3–4 weeks.

The beads made by extrusion and emulsion techniques were kept in peptone solution of pH 4 and 6 and exposed to 65 °C for 15 and 30 min (Table 5). Initially, the number of viable *B. longum* cells was in the range of 9.51–9.71 log_10_ cfu/g beads, and the number of viable cells was reduced after exposure to heat conditions. The type of co-encapsulating agent had no influence on the survival of the co-encapsulated cells at 15 and 30 min. The survival of co-encapsulated cells made by the extrusion and emulsion techniques was in the range of 8.32–8.47 and 7.34–7.52 log_10_ cfu/g, respectively, in the presence of the peptone solution of pH 6, and that of pH 4 was in the range of 7.43–7.67 and 6.40–6.57 log_10_ cfu/g, respectively, at 15 min. The slower diffusion of peptone solution in the *E. americana* matrix during heat treatment might have led to the better survival of the cells. Co-encapsulating agents used in this study offered little protection to *B. longum* at 30 min. This might happen due to the partial breakdown of *E. americana* bonds during heat processing. Free cells were very sensitive to heat treatment. The results indicated that excessive heat unfolded the structure of the bacterial cells, broke the linkage between monomeric units and destroyed the monomers, leading to bacterial death [23,30].

**Table 4:** Survival of microencapsulated *Bifidobacterium longum* with *Eleutherine americana* under refrigeration storage.

Microencapsulation	Time (Week)	Plate Count (log_10_ cfu/g)			
		pH			
		4		6	
		Extrusion Technique	Emulsion Technique	Extrusion Technique	Emulsion Technique
Co-encapsulated cells: alginate-*E. americana* extract	0	9.61 ± 0.11 ^aA^	9.57 ± 0.04 ^aA^	9.72 ± 0.15 ^aA^	9.80 ± 0.03 ^aA^
Co-encapsulated cells: alginate-oligosaccharide extract		9.50 ± 0.16 ^aA^	9.48 ± 0.13 ^aA^	9.61 ± 0.12 ^aA^	9.53 ± 0.10 ^aA^
Co-encapsulated cells: alginate-commercial fructo-oligosaccharides		9.58 ± 0.08 ^aA^	9.65 ± 0.17 ^aA^	9.65 ± 0.10 ^aA^	9.64 ± 0.05 ^aA^
Encapsulated cells: alginate		9.71 ± 0.12 ^aA^	9.52 ± 0.20 ^aA^	9.59 ± 0.04 ^aA^	9.58 ± 0.14 ^aA^
Non-encapsulated cells: free cells		9.50 ± 0.08 ^aA^	9.47 ± 0.09 ^aA^	9.60 ± 0.07 ^aA^	9.57 ± 0.18 ^aA^
Co-encapsulated cells: alginate-*E. americana* extract	2	8.42 ± 0.21 ^aA^	7.51 ± 0.02 ^aB^	9.54 ± 0.01 ^aA^	8.36 ± 0.07 ^aB^
Co-encapsulated cells: alginate-oligosaccharide extract		8.57 ± 0.11 ^aA^	7.62 ± 0.13 ^aB^	9.43 ± 0.03 ^aA^	8.52 ± 0.05 ^aB^
Co-encapsulated cells: alginate-commercial fructo-oligosaccharides		8.63 ± 0.13 ^aA^	7.54 ± 0.08 ^aB^	9.51 ± 0.10 ^aA^	8.60 ± 0.16 ^aB^
Encapsulated cells: alginate		7.70 ± 0.21 ^bA^	6.68 ± 0.16 ^bB^	8.65 ± 0.11 ^bA^	7.57 ± 0.10 ^bB^
Non-encapsulated cells: free cells		2.47 ± 0.17 ^cA^	2.47 ± 0.05 ^cA^	4.54 ± 0.01 ^cA^	4.54 ± 0.09 ^cA^
Co-encapsulated cells: alginate-*E. americana* extract	4	7.51 ± 0.07 ^bA^	6.64 ± 0.08 ^bB^	8.37 ± 0.05 ^bA^	7.52 ± 0.04 ^bB^
Co-encapsulated cells: alginate-oligosaccharide extract		8.60 ± 0.12 ^aA^	7.52 ± 0.11 ^aB^	9.21 ± 0.15 ^aA^	8.23 ± 0.12 ^aB^
Co-encapsulated cells: alginate-commercial fructo-oligosaccharides		7.56 ± 0.18 ^bA^	6.47 ± 0.18 ^bB^	8.37 ± 0.18 ^bA^	7.20 ± 0.13 ^bB^
Encapsulated cells: alginate		5.47 ± 0.23 ^cA^	4.62 ± 0.32 ^cB^	6.36 ± 0.21 ^cA^	5.31 ± 0.05 ^cB^
Non-encapsulated cells: free cells		0.00 ± 0.00 ^dA^	0.00 ± 0.00 ^dA^	0.00 ± 0.00 ^dA^	0.00 ± 0.00 ^dA^

Values are the means ± the standard deviation from duplicate determinations; different superscript uppercase letters (A, B) in the same row are significantly different among different microencapsulation techniques for each pH (*p* < 0.05); different superscript lowercase letters (a, b, c, d) in the same column are significantly different among different co-encapsulating agents for each time (*p* < 0.05).

**Table 5:** Survival of microencapsulated *Bifidobacterium longum* with *Eleutherine americana* under heat treatment.

Microencapsulation	Time (Week)	Plate Count (log_10_ cfu/g)			
		pH			
		4		6	
		Extrusion Technique	Emulsion Technique	Extrusion Technique	Emulsion Technique
Co-encapsulated cells: alginate-*E. americana* extract	0	9.63 ± 0.08 ^aA^	9.68 ± 0.06 ^aA^	9.53 ± 0.03 ^aA^	9.60 ± 0.06 ^aA^
Co-encapsulated cells: alginate-oligosaccharide extract		9.71 ± 0.11 ^aA^	9.62 ± 0.09 ^aA^	9.64 ± 0.11 ^a^	9.70 ± 0.05 ^aA^
Co-encapsulated cells: alginate-commercial fructo-oligosaccharides		9.54 ± 0.13 ^aA^	9.55 ± 0.10 ^aA^	9.58 ± 0.17 ^aA^	9.57 ± 0.11 ^aA^
Encapsulated cells: alginate		9.65 ± 0.21 ^aA^	9.51 ± 0.09 ^aA^	9.70 ± 0.16 ^aA^	9.63 ± 0.16 ^aA^
Non-encapsulated cells: free cells		9.56 ± 0.10 ^aA^	9.70 ± 0.21 ^aA^	9.64 ± 0.23 ^aA^	9.57 ± 0.17 ^aA^
Co-encapsulated cells: alginate-*E. americana* extract	15	7.43 ± 0.07 ^aA^	6.55 ± 0.07 ^aB^	8.32 ± 0.17 ^aA^	7.34 ± 0.05 ^aB^
Co-encapsulated cells: alginate-oligosaccharide extract		7.52 ± 0.05 ^aA^	6.40 ± 0.11 ^aB^	8.47 ± 0.05 ^aA^	7.41 ± 0.04 ^aB^
Co-encapsulated cells: alginate-commercial fructo-oligosaccharides		7.67 ± 0.15 ^aA^	6.57 ± 0.15 ^aB^	8.39 ± 0.11 ^aA^	7.52 ± 0.11 ^aB^
Encapsulated cells: alginate		5.49 ± 0.19 ^bA^	4.48 ± 0.10 ^bB^	6.48 ± 0.20 ^bA^	5.32 ± 0.23 ^bB^
Non-encapsulated cells: free cells		2.58 ± 0.18 ^cA^	2.58 ± 0.11 ^cA^	3.46 ± 0.06 ^cA^	3.46 ± 0.15 ^cA^
Co-encapsulated cells: alginate-*E. americana* extract	30	5.62 ± 0.06 ^aA^	3.61 ± 0.06 ^aB^	6.53 ± 0.06 ^aA^	5.71 ± 0.05 ^aB^
Co-encapsulated cells: alginate-oligosaccharide extract		5.74 ± 0.09 ^aA^	3.47 ± 0.05 ^aB^	6.41 ± 0.09 ^aA^	5.60 ± 0.07 ^aB^
Co-encapsulated cells: alginate-commercial fructo-oligosaccharides		5.60 ± 0.15 ^aA^	3.56 ± 0.14 ^aB^	6.38 ± 0.12 ^aA^	5.65 ± 0.12 ^aB^
Encapsulated cells: alginate		3.42 ± 0.11 ^bA^	1.27 ± 0.18 ^bB^	4.36 ± 0.15 ^bA^	2.79 ± 0.10 ^bB^
Non-encapsulated cells: free cells		0.00 ± 0.00 ^cA^	0.00 ± 0.00 ^cA^	0.00 ± 0.00 ^cA^	0.00 ± 0.00 ^cA^

Values are the means ± the standard deviation from duplicate determinations; different superscript uppercase letters (A, B) in the same row are significantly different among different microencapsulation techniques for each pH (*p* < 0.05); different superscript lowercase letters (a, b, c) in the same column are significantly different among different co-encapsulating agents for each time (*p* < 0.05).

## 4. Conclusions

*Eleutherine americana* extract and its oligosaccharide extract showed prebiotic properties, which included resistance to low pH and partial tolerance to human α-amylase. Microencapsulation of *Bifidobacterium longum* with the extract and oligosaccharide extract resulted in better survival than free cells after sequential incubation in simulated gastric and intestinal juices, refrigeration storage and heat treatment. The viability of microencapsulated *B. longum* with the extract and oligosaccharide extract made by the extrusion technique was higher than that of the emulsion technique after exposure to adverse conditions. We demonstrated the possibility of using microencapsulated *Bifidobacterium* in some technological processes, such as during the manufacturing of functional foods that need to be subjected to a heat treatment, such as pasteurization. Further studies are warranted in food products before potential applications for consumers.
